# LiFePO_4_ microcrystals as an efficient heterogeneous Fenton-like catalyst in degradation of rhodamine 6G

**DOI:** 10.1186/1556-276X-9-276

**Published:** 2014-05-30

**Authors:** Zhan Jun Li, Ghafar Ali, Hyun Jin Kim, Seong Ho Yoo, Sung Oh Cho

**Affiliations:** 1 Department of Nuclear and Quantum Engineering, Korea Advanced Institute of Science and Technology (KAIST), Daejeon 305701, Republic of Korea

**Keywords:** Fenton-like catalyst, LiFePO_4_, Rhodamine 6G, Advanced oxidation

## Abstract

We present a novel heterogeneous Fenton-like catalyst of LiFePO_4_ (LFP). LFP has been widely used as an electrode material of a lithium ion battery, but we observed that commercial LFP (LFP-C) could act as a good Fenton-like catalyst to decompose rhodamine 6G. The catalytic activity of LFP-C microparticles was much higher than a popular catalyst, magnetite nanoparticles. Furthermore, we found that the catalytic activity of LFP-C could be further increased by increasing the specific surface area. The reaction rate constant of the hydrothermally synthesized LFP microcrystals (LFP-H) is at least 18 times higher than that of magnetite nanoparticles even though the particle size of LFP is far larger than magnetite nanoparticles. The LFP catalysts also exhibited a good recycling behavior and high stability under an oxidizing environment. The effects of the experimental parameters such as the concentration of the catalysts, pH, and the concentration of hydrogen peroxide on the catalytic activity of LFP were also analyzed.

## Background

Advanced oxidation processes (AOPs) based on highly oxidative hydroxyl radicals have been developed to degrade organic pollutants into harmless water and carbon dioxide [1-3]. Various organic pollutants such as organic dyes [4], microcystins [5], phenol and its derivatives [6], biological-resistant pharmaceuticals [7], and landfill leachate [8] can be decomposed through AOPs. Fenton process, which uses dissolved ferrous salt as a homogeneous catalyst to produce hydroxyl radicals from hydrogen peroxide, is one of the pioneering works in AOPs. However, homogeneous Fenton catalysts exhibit good performance only when pH < 3.0 because high acidic environment is necessary to prevent the precipitation of ferrous and ferric ions [8-10]. Furthermore, homogeneous Fenton catalysts can hardly be recycled [11,12], and a large amount of iron sludge is generated in the process. To overcome these drawbacks, recyclable heterogeneous Fenton-like catalysts have been developed, including Fe_3_O_4_[13,14], BiFeO_3_[15], FeOCl [16], LiFe(WO_4_)_2_[17]_,_ iron-loaded zeolite [4,18], iron-containing clay [19], and carbon-based materials [20,21]. Comparing to homogeneous Fenton catalyst, these heterogeneous Fenton-like catalysts can degrade the organic pollutants in a wider pH range [11,12,15]. Moreover, the heterogeneous catalysts based on particles can be recycled by filtration, precipitation, centrifuge, and magnetic field [4,10,11]. However, the catalytic activities of the heterogeneous Fenton-like catalysts were comparatively low for the practical applications [12,15,16]. Nanometer-sized catalysts have been tried to improve the activities, but nano-catalysts require complicated processes for synthesis, prevention of nanoparticle agglomeration, and size/shape control. In addition, recycle of nano-catalysts by filtration, precipitation, and centrifuge methods is difficult. Magnetite nanoparticles can be easily recycled by using a magnetic field and thus these are widely studied as a Fenton-like catalyst, but the catalytic activities are still not satisfactory for practical applications [12,13,22,23]. Therefore, it is crucial to develop novel efficient heterogeneous Fenton-like catalysts.

Herein, we report a novel Fenton-like catalyst, LiFePO_4_ (LFP). LFP is usually used as an electrode material of a lithium ion battery [24,25]. Interestingly, we found that commercialized LFP particles with micrometer sizes showed much better catalytic activity in degrading rhodamine 6G (R6G) than magnetite nanoparticles. Moreover, the catalytic activities of LFP microcrystals could be further improved by decreasing the particle sizes.

## Methods

### Materials and synthesis

Lithium hydroxide, ammonium Fe (II) sulfate hexahydrate, phosphoric acid, commercial LFP (abbreviated as LFP-C), and R6G are all purchased from Sigma-Aldrich (St. Louis, MO, USA) and used as received. Magnetite nanoparticles were synthesized according to a reported co-precipitation method [26]. LFP microcrystals (abbreviated as LFP-H) were synthesized using a hydrothermal method [27]. Briefly, ammonium Fe (II) sulfate hexahydrate (5.882 g) and phosphoric acid (1.470 g) were dissolved into 40 mL of water. Lithium hydroxide (1.890 g) was also dissolved into 10 mL of water. And then, these two solutions were quickly mixed under vigorous magnetic stirring at room temperature. After stirring for 1 min, the mixture was poured into a 60-mL Teflon-lined autoclave. The autoclave was heated in a furnace at 220°C for 3 h. The as-synthesized LFP-H can be easily separated by using a filter paper. After being washed by 95% ethanol for three times, the LFP-H particles were air-dried at 60°C for 24 h.

### Degradation experiments

R6G was chosen as a model contaminant. The oxidation decolorization experiments of R6G were carried out in 50 mL conical flasks. Unless otherwise specified, the experiments were performed at 20°C. Briefly, a certain amount of catalysts were added into 50 mL R6G aqueous solution with a concentration of 30 μg/mL. The pH was adjusted by diluted sulfate acid and sodium hydroxide. The suspension was stirred for 1 h to achieve the adsorption/desorption equilibrium between the solid catalyst and the solution. The concentration of R6G after the equilibrium was taken as the initial concentration (*C*_0_). The degradation started just after an addition of hydrogen peroxide (30%) under stirring. Samples (1 mL) were taken from the reaction flask at a given time interval. The oxidation reaction was stopped by adding 100 μL of 1 M sodium thiosulfate solution. The catalyst was separated from the sample by a centrifuge at 10,000 rpm for 5 min. The concentration of the supernatant (*C*) was detected by using a UV-visible spectrometer after a water dilution of three times.

### Characterization

X-ray powder diffraction (XRD) was performed on a X-ray diffractometer (Rigaku, D/MAX-2500, Shibuya-ku, Japan) with Cu Kα radiation (*λ* = 1.5418 Å). The morphologies of the samples were observed using a field-emission scanning electron microscopy (FESEM, Hitachi, S-4800, Chiyoda-ku, Japan) and a high-resolution transmission electron microscope (HRTEM, Philips, Tecnai F20, Amsterdam, The Netherlands) at an accelerating voltage of 200 kV. The N_2_ adsorption/desorption isotherms were performed on a full-automatic physical and chemical adsorption apparatus (Micromeritics, TriStar II 3020, Norcross, GA, USA).

## Results and discussion

### Morphologies and catalytic activities of the as-synthesized magnetite and LFP-C

**Figure 1:**
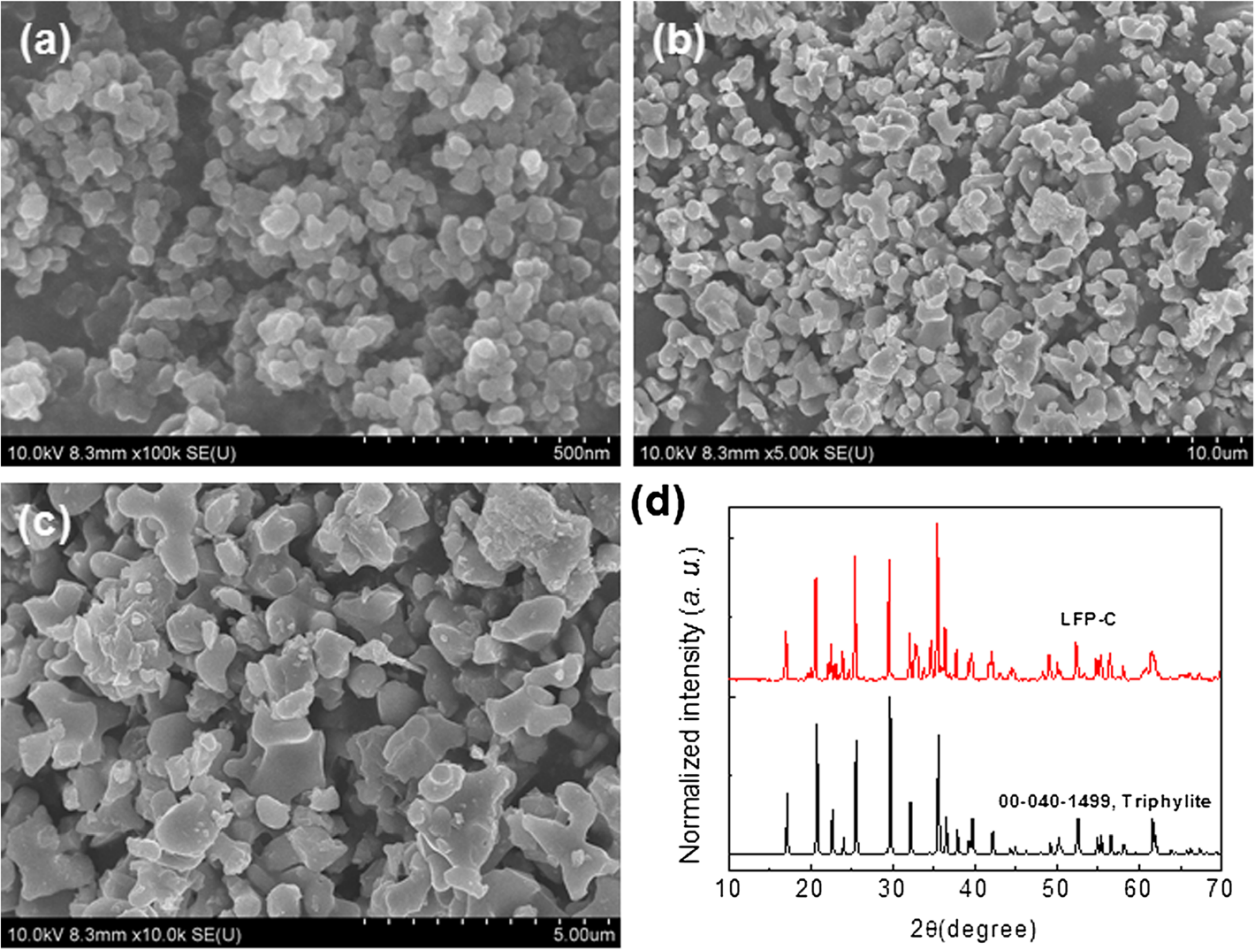
**FESEM images and XRD pattern.** FESEM images of the as-synthesized magnetite nanoparticles **(a)** and **(b, c)** the LFP-C particles. **(d)** XRD pattern of the LFP-C particles.

Magnetite nanoparticles were widely studied as a Fenton-like catalyst due to the ferrous element, and we chose magnetite nanoparticles as a reference catalyst to evaluate the catalytic activity of LFP [9,10]. In our experiment, magnetite nanoparticles were synthesized by co-precipitation of ferrous and ferric solutions with a molar ratio of Fe(III)/Fe(II) of 2:1 at 80°C [27]. The FESEM result indicates that the as-synthesized magnetite nanoparticles have a quite small average particle size of approximately 50 nm with a narrow size distribution (Figure 1a). In contrast, the as-received LFP-C has much bigger particle size than the as-synthesized magnetite. The FESEM images of LFP-C shows that the commercial product of LFP-C has particle sizes from approximately 1 to approximately 4 μm with irregular morphologies (Figure 1b,c). The XRD analysis of LFP-C indicates that the commercial LFP-C is composed of a triphylite crystal phase (JCPDS card no. 00-040-1499) (Figure 1d).

**Figure 2:**
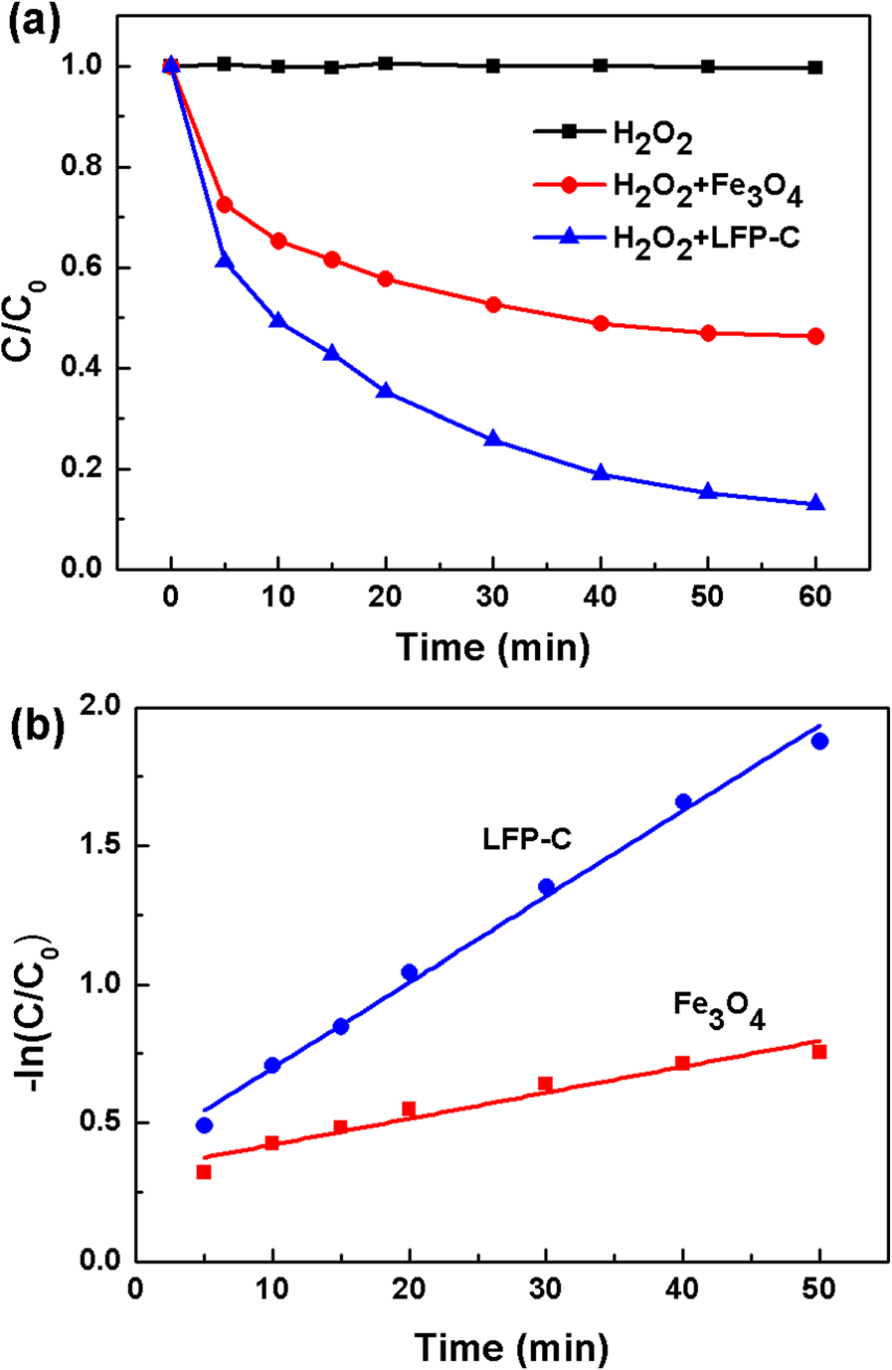
**Degradation behavior and kinetic analysis. (a)** Degradation behavior of R6G by the magnetite nanoparticles and the LFP-C catalysts. **(b)** Kinetic analysis of the degradation curves. The concentrations of the LFP-H and H_2_O_2_ (30%) were 3 g/L of and 6 mL/L, respectively, and pH of the solution was 7.

In order to evaluate the potential of LFP-C as heterogeneous Fenton-like catalyst, oxidative degradation experiments of R6G with hydrogen peroxide were performed. The degradation behaviors of R6G and magnetite catalysts were shown in Figure 2a. The concentration of the catalysts and hydrogen peroxide were 3 g/L and and 6 mL/L, respectively, and the pH of R6G solution was 7. The degradation efficiency of approximately 53.7% was achieved with magnetite nanoparticles after 1 h reaction. However, LFP exhibited the efficiency of 86.9% after 1 h, which is much higher than that of magnetite nanoparticles. This is somewhat surprising because the particle size (a few μm) of LFP is much larger than that (approximately 50 nm) of magnetite nanoparticles: larger particles lead to smaller surface area for the interfacial catalytic reaction, thereby worse catalytic activity. The kinetic analysis of the degradation curves (Figure 2b) indicates that the heterogeneous catalytic degradation of R6G approximately followed a pseudo first-order reaction, which may be expressed as − ln (*C*/*C*_0_) = *k t* + *y*, where *k* is the apparent rate constant (min^−1^), *t* is the reaction time (min), *y* is a constant, and *C*_0_ and *C* are the concentration of R6G (mg/L) at time of *t* = 0 and the sampling time, respectively. The *k* value (0.03) of LFP-C is three times higher than that of magnetite nanoparticles (0.009). Considering the difference in the particle sizes, we can conclude that LFP-C has much higher catalytic activity than magnetite.

### Morphology and catalytic activity of the as-synthesized LFP-H

**Figure 3:**
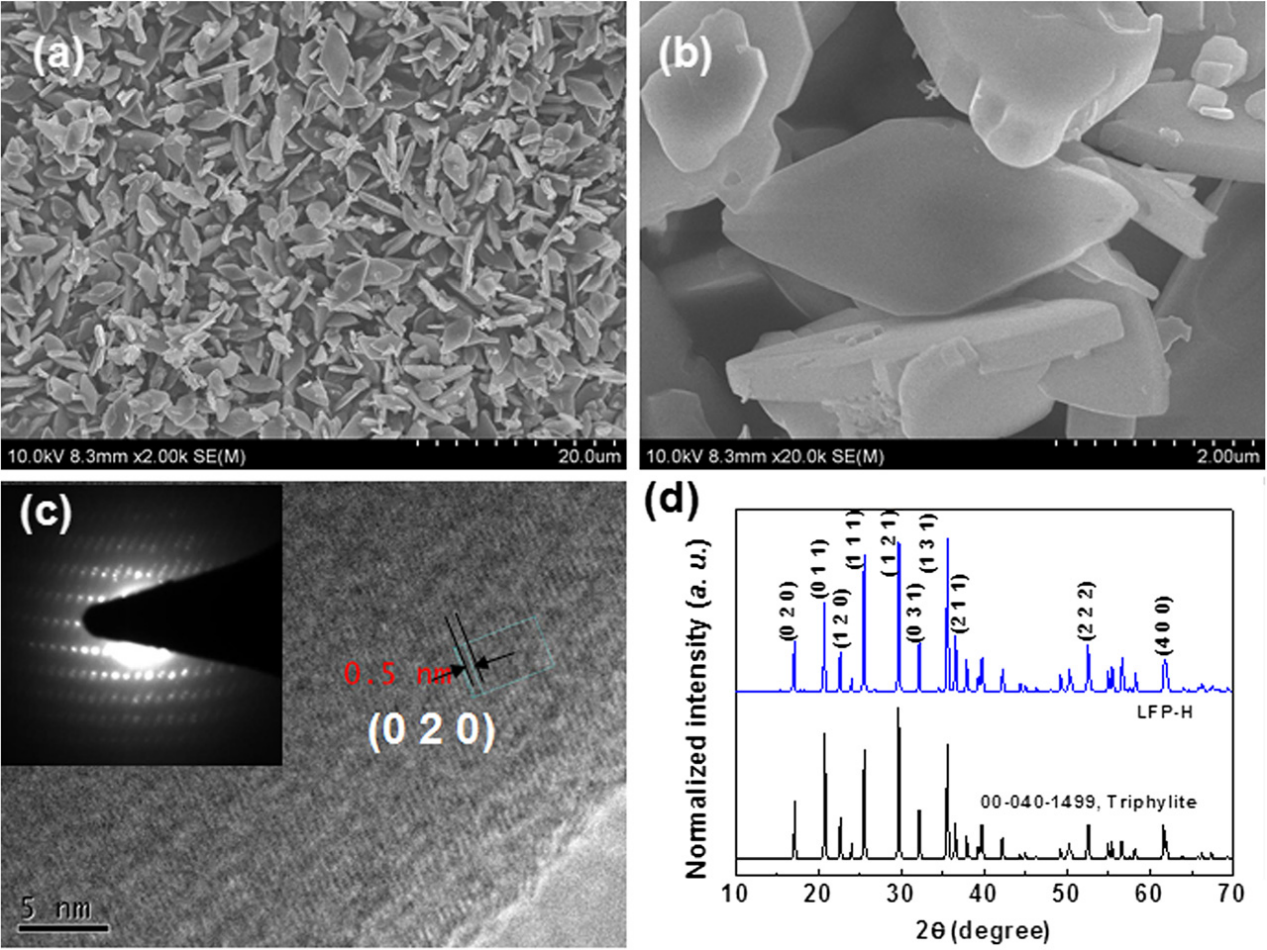
**FESEM, HRTEM, SAED, and XRD patterns. (a, b)** FESEM images, **(c)** HRTEM image and the SAED pattern, and **(d)** XRD pattern of the as-prepared LFP-H particles.

As shown in Figure 1b,c, LFP-C has irregular morphology and big particle size, which suggests that the catalytic performance of LFP might be improved by adjusting its morphology and particle size. Therefore, we tried to synthesize LFP with regular morphologies and bigger specific surface area using a hydrothermal method [27]. We observed that higher heating rate is crucial for the formation of regular microcrystals. When the temperature of the autoclave was increased from room temperature to 220°C with a heating rate of (approximately 4°C/min), only irregular LFP particles were created [Additional file 1: Figure S1a,b]. Even though the heating duration was increased to 24 h at 220°C, no significant improvement in the morphologies was observed. However, when the heating rate was dramatically increased by inserting an autoclave into a pre-heated oven maintained at 220°C, regular LFP particles with a rhombus-like plate morphologies were prepared (Figure 3, hereafter, the particles are expressed as LFP-H). The LFP particles had thicknesses of 200 to 500 nm and edge lengths of 2 to 4 μm. The HRTEM image and the SAED pattern indicate a good crystallinity of the LFP-H (Figure 3c). The XRD pattern reveals that LFP-H particles are triphylite (JCPDS card no. 00-040-1499) without any observable impurities (Figure 3d).

**Figure 4:**
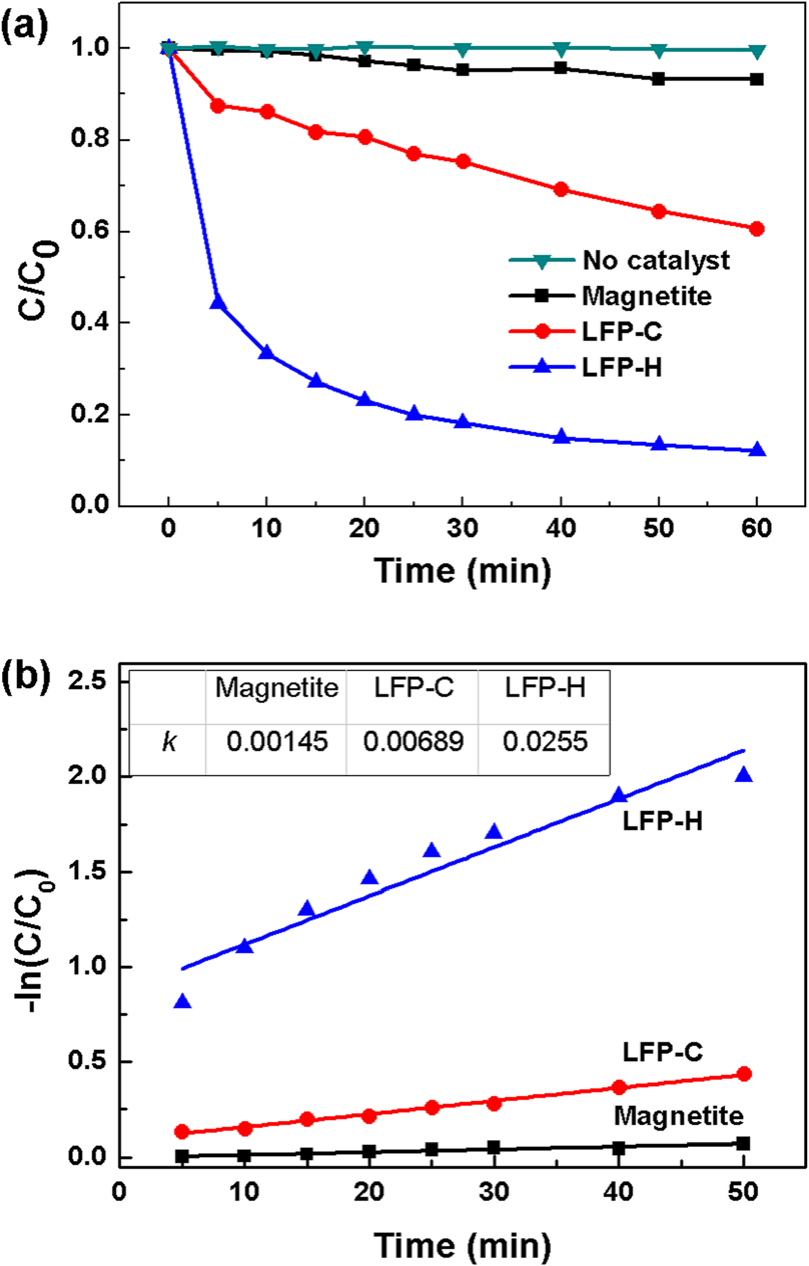
**Degradation behavior and kinetic analysis. (a)** Degradation behavior of R6G by the LFP-H catalyst. **(b)** Kinetic analysis of the degradation curves. The concentrations of the LFP-H and H_2_O_2_ (30%) were 1 g/L of and 1 mL/L, respectively, and pH of the solution was 7.

When the catalytic degradation experiments of R6G using the fabricated LFP-H particles were carried out, we observed that the activity of the as-synthesized LFP-H is so high that R6G is completely decomposed in a few min [Additional file 1: Figure S2, the experimental condition was the same with Figure 2]. As a result, the degradation curve cannot be measured accurately, and thus, the concentration of the catalyst and hydrogen peroxide was decreased to 1 g/L, and 1 mL/L, respectively, which is beneficial to reduce the cost of the degradation process. Even at this condition, the LFP-H exhibited a degradation efficiency of 87.8% for R6G. In comparison, magnetite nanoparticles and LFP-C showed degradation efficiencies of only 6.8% and 39.3%, respectively (Figure 4a). The kinetic analysis of the degradation curves (Figure 4b) indicates that the oxidation reaction catalyzed by LFP-H can also be fitted by a pseudo first-order reaction. The ratio of *k* value of LFP-H/LFP-C/magnetite is 18/5/1, indicating that LFP-H is much better Fenton-like heterogeneous catalyst than LFC-C and magnetite. Higher activity of LFP-H than LFP-C is mainly attributed to higher surface area. Brunauer-Emmett-Teller (BET) measurement [Additional file 1: Figure S3] shows that the specific surface areas of LFP-C and LHP-H were 1.51 and 3.36 m^2^/g, respectively. The average size of the as-synthesized LFP-H particles is a few micrometers. Therefore, the catalytic activity of LFP-H can be further improved by using nanostructured LFP-H particles because the specific surface area of the particles can be increased by decreasing the particle size.

### Effects of the experimental parameters on the catalytic activity of LFP-H

**Figure 5:**
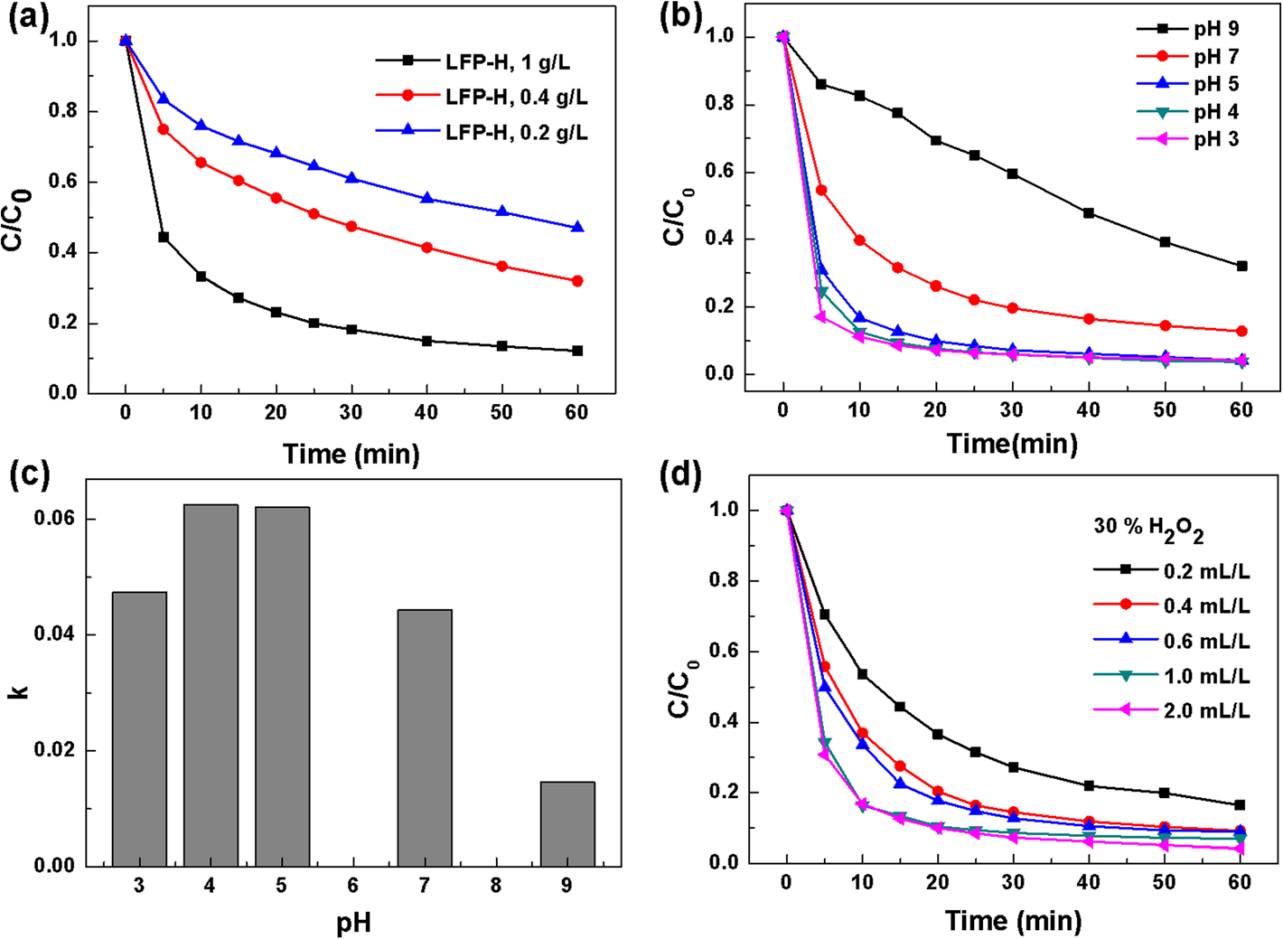
**Oxidation decolorization experiments of R6G. (a)** at different concentration of LFP-H particles with fixed concentration of 1 mL/L H_2_O_2_ (30%) and pH of 7, **(b, c)** at different pH with fixed LFP-H concentration of 1 g/L LFP-H and 1 mL/L H_2_O_2_ (30%), and **(d)** at different H_2_O_2_ concentration with fixed concentration of 1 g/L LFP-H and pH of 5.

Systematic experiments to investigate the effects of the concentration of the catalysts, pH, and the concentration of hydrogen peroxide on the catalytic activity were carried out. First, when the concentration of LFP-H particles was reduced from 1 to 0.2 g/L at pH of 7, the degradation efficiency of R6G decreased from 87.8% to 53.0% after 1 h and *k* value decreased from 0.026 to 0.011 (Figure 5a). Second, LFP-H particles worked as a moderately good catalyst over a broad range of pH from 3 to 9 (Figure 5b,c). Highest catalytic activities were observed at weak acidic conditions of pH = 4 to 7, and the activities were decreased at high acidic condition (pH = 3) and weak basic condition (pH = 9); the ratio of *k*(pH = 3)/*k*(pH = 4)/*k*(pH = 5)/*k*(pH = 7)/k(pH = 9) is approximately 3.2:4.3:4.3:3/1, respectively. Third, the catalytic activity increased with the increase in the concentration of hydrogen peroxide below 1 mL/L but did not change so much above 1 mL/L (Figure 5d). The degradation efficiency of R6G was almost the same after 1 h when the hydrogen peroxide concentration was above 0.4 mL/L.

### Catalytic behavior of the recycled LFP-H

**Figure 6:**
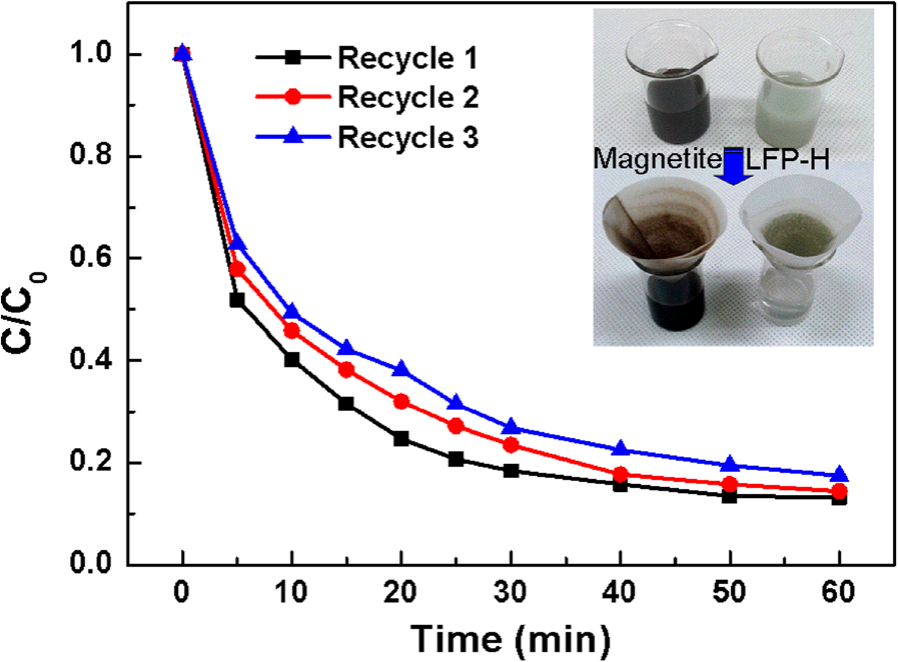
Catalytic behavior of the recycled LFP-H particles.

One of the most important advantages of heterogeneous catalysts is their capability of reuse [1,5,6,18]. The LFP-H catalyst can be easily recycled by filtration due to the relative big particle size, while the magnetite nanoparticles are difficult to be recycled by filtration, as shown in the inset of Figure 6. This fact is one of the advantages of LFP-H microcrystals. LFP-H still showed a high Fenton-like catalytic activity even after three times of recycle (Figure 6). A slight decrease in the degradation rate of R6G occurred with the increase in the recycle number. We observed that the color of the LFP-H microcrystals slightly changed from light gray to dark gray, indicating that oxidation of LFP-H occurred, possibly Fe(II) in LFP-H was transformed to Fe(III) [28]. The slow oxidation of LFP-H during oxidation of R6G might be the reason of the slight decrease in the catalytic activity. In addition, we observed that almost no color was changed when LFP-H was stored in an oven at 60°C for one week, indicating that LFH-H is very stable against air oxidation. This high stability of LFP-H in ambient atmosphere is a good advantage for practical application.

## Conclusions

We report that LFP, which is widely used as an electrode material of a lithium ion battery, can act as an excellent heterogeneous Fenton-like catalyst. The LFP microparticles exhibited much better catalytic activities to decompose R6G than a popular Fenton-like catalyst of magnetite nanoparticles. The LFP microparticles also showed a good recycling behavior as a Fenton-like catalyst. In addition, the catalytic activities of LFP can be improved by increasing the specific surface area, suggesting that the catalytic activity of LFP can be further improved if nanostructured LFP particles can be properly synthesized. We believe that LFP can be practically used as a catalyst due to its high catalytic activity and a good recycling behavior. Furthermore, LFP may open new application fields if the catalytic property of LFP is combined with the conventional properties that are useful as an electrode of a battery.

## Competing interests

The authors declare that they have no competing interests.

## Authors' contributions

ZJL conceived the original idea, carried out most of the experiments, and drafted the manuscript. GA helped to design the oxidation experiments, analyzed the data, and participated in the writing of the manuscript. HJK carried out the morphology characterization. SHY helped to design the experiment devices. SOC supervised the research process and provided constructive opinions to improve the quality of the research. All authors read and approved the final manuscript.

## Supplementary Material

Additional file 1: Figure S1 FESEM images. (a) FESEM images of LFP synthesized by hydrothermal method with a slow heating rate of approximately 4°C/min. (b) Magnified FESEM image of (a). **Figure S2.** Compare of LFP-H and LFP-C in catalytic degradation of R6G. Conditions: 3 g/L of catalyst, 6 mL/L of H_2_O_2_ (30%), pH=7. **Figure S3.** N_2_ adsorption/desorption isotherms of LFP-C and LFP-H. Click here for file
