# Supplementary material for: LiFePO4 microcrystals as an efficient heterogeneous Fenton-like catalyst in degradation of rhodamine 6G
Source: Nanoscale Res Lett. 2014 May 30;9(1):276. doi: 10.1186/1556-276X-9-276 (PMC4051962; doi:10.1186/1556-276X-9-276)
Supplement: Additional file 1: Figure S1 — FESEM images. (a) FESEM images of LFP synthesized by hydrothermal method with a slow heating rate of approximately 4°C/min. (b) Magnified FESEM image of (a). Figure S2. Compare of LFP-H and LFP-C in catalytic degradation of R6G. Conditions: 3 g/L of catalyst, 6 mL/L of H2O2 (30%), pH=7. Figure S3. N2 adsorption/desorption isotherms of LFP-C and LFP-H. [file 1556-276X-9-276-S1.doc]

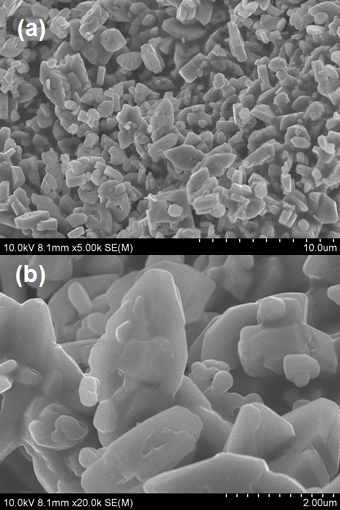


Additional file 1: Figure S1. (a) FESEM images of LFP synthesized by hydrothermal method with a slow heating rate of ~4 ^o^C/min. (b) Magnified FESEM image of (a).

##

## Additional file 1: Figure S2. Compare of LFP-H and LFP-C in catalytic degradation of R6G. Conditions: 3 g/L of catalyst, 6 mL/L of H_2_O_2_ (30%), pH=7.

##

## Additional file 1: Figure S3. N_2_ adsorption/desorption isotherms of LFP-C and LFP-H.
